# Identification of a novel mutation in the *NTF4* gene that causes primary open-angle glaucoma in a Chinese population

**Published:** 2010-08-15

**Authors:** Eranga N. Vithana, Monisha E. Nongpiur, Divya Venkataraman, Stephanie H. Chan, Jagadeesh Mavinahalli, Tin Aung

**Affiliations:** 1 Singapore Eye Research Institute, 11 Third Hospital Avenue, Singapore; 2 Department of Ophthalmology, National University of Singapore, Lower Kent Ridge Road, Singapore; 3 Bioinformatics Institute, Matrix #07-01, 30 Biopolis Street, Singapore; 4 Singapore National Eye Centre, 11 Third Hospital Avenue, Singapore

## Abstract

**Purpose:**

Neurotrophin-4 protein (NT-4) plays a role in the protection of retinal ganglion cells by activating tyrosine kinase B (TrkB) receptors. A recent study identified mutations within the neurotrophin-4 (*NTF4*) gene to account for 1.7% of primary open-angle glaucoma (POAG) in Europeans. The aim of this study was to investigate the frequency of *NTF4* mutations in Chinese POAG patients.

**Methods:**

One hundred-seventy-four Chinese subjects with POAG and 91 normal Chinese subjects were recruited. POAG was defined by the presence of glaucomatous optic neuropathy, open angles on gonioscopy, and absence of secondary causes of glaucoma. The single coding exon of *NTF4* was PCR amplified and subjected to bidirectional sequencing in all subjects.

**Results:**

The mean age of POAG patients was 66.0±13.0 years (range 25–96 years) and that of controls was 67.1±4.6 years (range 60–85 years). We identified a novel *NTF4* missense mutation substituting leucine by serine at codon 113 (Leu113Ser) caused by a c.338T>C mutation in a single patient with unilateral POAG, who presented with a baseline intraocular pressure of 25 mmHg, a vertical cup-to-disc ratio of 0.9 and an inferior hemifield defect in the affected eye. Structural analysis indicated that the Leu113Ser mutation is likely to alter the NT-4 protein structure near the TrkB binding site and disrupts the formation of the NT-4-TrkB complex required for the activation of TrkB.

**Conclusions:**

Identification of a single mutation in our study suggests that *NTF4* mutations are a rare cause of POAG (0.6%, 95%CI 0.02%–3.16%) in Chinese people.

## Introduction

Glaucoma is the leading cause of irreversible blindness worldwide and affects about 70 million people [1,2], with primary open angle glaucoma (POAG) being the most prevalent form of the disease. Asymptomatic in the early stages, POAG is characterized by a progressive optic neuropathy and loss of retinal ganglion cells (RGCs) [3] resulting in corresponding loss of the visual field. The intraocular pressure (IOP) is often but not invariably elevated and is considered an important risk factor contributing to visual loss [4,5].

POAG is a complex disorder where both genetic and environmental factors have been shown to play a part in its development [6]. Several studies have demonstrated a significant familial heritable basis where a large proportion of patients had a positive family history [4,7]. To date, 3 genes have been identified for POAG; myocilin (*MYOC* or GLC1A) [8], optineurin (*OPTN* or GLC1E) [9], and WD40-repeat 36 (*WDR36* or GLC1G) [10], and also associated with it are a large number of genetic variants including those in apolipoprotein E (*APOE*) [11], optic atrophy 1 (*OPA1*) [12], and cytochrome P4501B1 (*CYP1B1*) [13]. Although the exact role of *MYOC* and *OPTN* in the pathogenesis of glaucoma is unclear, studies have shown that myocilin may have a role in trabecular meshwork homeostasis [14], while optineurin is implicated in the neuroprotection of RGCs by reducing their susceptibility to hydrogen peroxide-induced cell death [15]. However, mutations in these known genes account for only a small fraction of POAG patients.

The complex nature of the disease phenotype combined with the vast genetic heterogeneity suggests the involvement of multiple pathological processes and molecular pathways in POAG causation. Recently, mutations in a new gene, neurotrophin-4 (*NTF4*) located in chromosome 19q13.33 [16], have been implicated in 1.7% of POAG patients of European origin [17]. It has been shown that neurotrophin 4 protein (NT-4) plays a role in the protection of the RGCs by activating the tyrosine kinase B (TrkB) receptor present in these cells [18,19]. Low levels of TrkB expression leads to a progressive loss of RGCs [20], a situation that also occurs during the course of the disease process in glaucoma, which is reflected as glaucomatous visual field damage. Furthermore, mutations in *NTF4* resulted in an in-vitro impairment of TrkB signaling as well as neuronal growth [17]. The recent identification in glaucoma patients, of seven different heterozygous *NTF4* mutations reveals a crucial role of the neurotrophin signaling system in preventing neural degeneration, thus supporting the possibility of another pathway in the pathogenesis of glaucoma.

The purpose of our study was to investigate the spectrum and frequency of *NTF4* mutations in our panel of Chinese patients with POAG. In this paper we describe a rare novel mutation of *NTF4* located in the conserved region of the protein sequence.

## Methods

### Patients

Study subjects of Chinese ethnicity were recruited from clinics at the Singapore National Eye Centre. Written, informed consent was obtained from all subjects and the study had the approval of the Ethics Committee of the Singapore Eye Research Institute and was performed according to the tenets of the Declaration of Helsinki.

All subjects underwent a standardized ophthalmic examination that included best corrected Snellen visual acuity, slit-lamp examination (Model BQ 900; Haag-Streit, Bern, Switzerland), stereoscopic disc examination with a 78-diopter lens (Volk Optical Inc., Mentor, OH), gonioscopy, and IOP measurement by Goldmann applanation tonometer (Haag-Streit, Koniz, Switzerland). POAG was defined by the following criteria: the presence of glaucomatous optic neuropathy (defined as loss of neuroretinal rim with a vertical cup: disc ratio of >0.7 or an inter-eye asymmetry of >0.2, and/or notching attributable to glaucoma) with compatible visual field loss, open angles on gonioscopy, and absence of secondary causes of glaucomatous optic neuropathy such as a period of steroid administration, or uveitis. A glaucomatous visual field defect was defined if the following were found: (1) glaucoma hemifield test (GHT) outside normal limits, (2) a cluster of 3 or more, non-edge, contiguous points on the pattern deviation plot, not crossing the horizontal meridian with a probability of <5% being present in age-matched normals (one of which is <1%) and (3) PSD <0.05; these were repeatable on two separate occasions. All normal control subjects had IOP<21 mmHg with open angles, healthy optic nerves, normal visual fields and no family history of glaucoma.

### Mutation screening

Genomic DNA was extracted from peripheral blood leukocytes from all the subjects. The coding regions of *NTF4* were amplified by polymerase chain reaction (PCR) with custom-designed primers. Because of it size, the coding exon of *NTF4* was divided into two overlapping PCR fragments amplified by primers NTF4–2F1–5′-ATT AGA GGT GTG GGG CAC AG-3′; NTF4–2R1–5′-CAG CCA CTG ACT GCA TCG-3′ and NTF4–2F2–5′- GCC CCC TCT GCT CTT CCT-3′ NTF4–2R2–5′-TTT GAT GAG TTC CCA AAC TGG-3′. PCR reactions were performed in a 50-μl mixture containing 10 mM Tris-HCL (pH 8.9), 50 mM KCL, 1.5 mM MgCl_2_, 200 μM each deoxyribose nucleoside triphosphate, 25 picomoles of each primer (100 μM concentration), 0.2 μl of HotstarTaq DNA polymerase (Qiagen, GmbH, Hilden, Germany) and 4 μl of genomic DNA (50–100 ng). Thermal cycling was performed (DNA Thermocycler 9700; Applied Biosystems, Foster City, CA.) under the following conditions: initial denaturation for 15 min at 95 °C; 40 cycles of 95 °C for 30 s, annealing variable temperature 58 °C to 62 °C for 30 s, and extension 72 °C for 30 s; and a final extension 72 °C for 5 min. The PCR products were analyzed on an agarose gel to confirm the product size and purified using GFX PCR clean up columns (GFX; Amersham, Piscataway, NJ). Sequence variations were identified by automated bi-directional sequencing using BigDye Terminator Mix v3.1; according to manufacturer’s protocols (Applied Biosystems). Samples were denatured at 96 °C for 1 min, then cycled 25 time at 96 °C for 10 s, 50 °C for 5 s, 60 °C for 4 min. Primers for sequence reactions were the same as those for the PCR reaction. Removal of unincorporated nucleotides and purification of PCR BigDye products were performed by ethanol precipitation at 4 °C. The samples were resuspended in hi-di formamide before sequencing. An automated DNA sequencer (ABI PRISM 3100; Applied Biosystems) was used. Sequence alterations were recorded based on *NTF4* cDNA sequence with +1 corresponding to the A of the ATG translation initiation codon in reference sequence NM_006179 (version NM_006179.4). The known POAG genes *MYOC, OPTN*, and *WDR36* were also screened, in a single POAG patient harboring a *NTF4* sequence variation, by PCR and direct sequencing using previously described conditions [21,22].

### Molecular modeling

The crystal structure of the NTF4-TrkB complex (PDB code: 1HCF) [23] was used as a template to model the Leu113Ser mutation. The Leu113 residue of the wild type protein was substituted with Ser113 using the PyMOL program (The PyMOL Molecular Graphics System [2002], DeLano Scientific, Palo Alto, CA) and subjected to energy minimization, using the Sander module of Amber simulation package [24] with force field ff94, comprising 50 steps of steepest descent with a cut off for non-bonded interactions of 8 Å.

## Results

### Mutation analysis

One hundred-seventy-four Chinese patients with POAG and 91 Chinese control subjects were recruited in the study. The mean age of the patients was 66.0±13.0 years (range 25–96 years) and that of the controls was 67.1±4.6 years (range 60–85 years).

**Figure 1:**
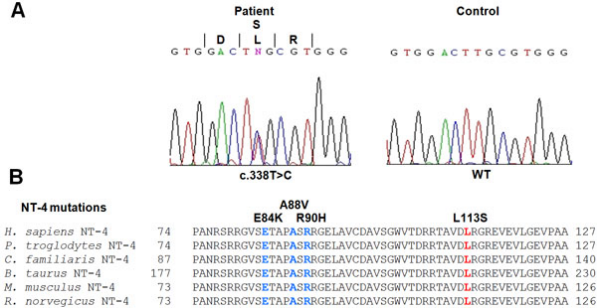
The mutation in *NTF4* identified in the Chinese POAG patient. **A**: Sequence electropherogram of the *NTF4* heterozygous mutation c.338T>C (Leu113Ser) identified in a sporadic POAG case (left). Wild type sequence from an unaffected control individual is shown to the right for comparison. **B**: Partial sequence (residues 74 to 127) of human (*H. sapiens*) NT-4 polypeptide compared with orthologs from other mammalian species showing the conservation of the L113 residue as well it’s position in relation to previously reported mutations E84K, A88V and R90H.

Screening of *NTF4* in the patients did not reveal any of the previously identified pathogenic mutations associated with POAG, namely, C7Y, E84K, A88V, R90H, R206W, R206Q, and R209G [17]. We identified a novel missense mutation substituting leucine by serine at codon 113 (Leu113Ser) caused by a c.338T>C mutation in exon 2 in a single patient with unilateral POAG (Figure 1A). To exclude the involvement of the known POAG genes *MYOC, OPTN*, and *WDR36*, we also re-sequenced all exons and adjacent splice sites of these genes in this patient. We did not find any deleterious protein altering sequence changes in *MYOC*, *OPTN*, and *WDR36*, indicating that these genes are unlikely to be the cause of POAG in this patient. However, we did identify three previously reported *OPTN* polymorphisms (c.412G>A [T34T], IVS7+24G>A, and IVS15–48C>A) and three *WDR36* polymorphisms (c.790A>G [I264V], IVS3–113G>A, and IVS16–30A>G) in this patient. The c.338T>C mutation in *NTF4* was not found in any of our 91 normal controls and was also located in the highly conserved mature domain of the NT-4 protein (Figure 1B). We also did not find this gene to be polymorphic in our patient and control sample sets, as no other sequence change was identified besides the c.338T>C change.

The patient concerned was a 70-year-old lady first diagnosed with unilateral POAG at age 67. At presentation, examination of the affected eye revealed a pale disc with a vertical cup-to-disc ratio (VCDR) of 0.9, IOP of 25 mmHg and an inferior hemifield visual field defect, while the fellow unaffected eye had a VCDR of 0.5 with healthy neuro-retinal rims, IOP of 15 mmHg, and normal visual field. Gonioscopy revealed 360° of open drainage angles in both eyes. She underwent a combined phacoemulsification and trabeculectomy with mitomycin C application, and was on regular follow-up. About 20 months post-surgery, the IOP increased due to failing bleb function, and she was started on medications to control the IOP. At final follow up, the patient is still on medications with the IOP within acceptable limits. Systemically, she has arterial hypertension for which she is on treatment. Familial segregation could not be determined as she was unmarried, and her parents were deceased and not known if they were also affected. Her 3 siblings have not as yet been diagnosed to have glaucoma, and they refused genetic analysis.

### Protein modeling of the mutation

**Figure 2:**
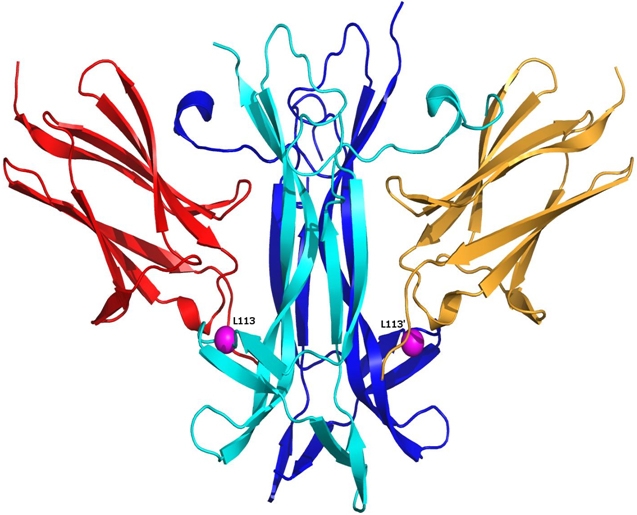
Location of the Leu113Ser mutation in the crystal structure of the NT-4-TrkB complex. The two chains of the dimeric NT-4 are in cyan and blue, respectively. The alpha carbon of the mutated residue is shown as a magenta sphere, with the prime symbol denoting the residue of the second subunit. TrkB domains are shown in orange and red color. The Leu113 residue is located close to the TrkB binding site.

**Figure 3:**
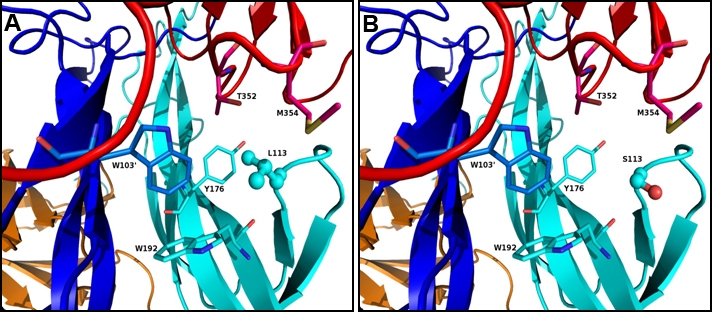
Immediate neighboring residues of Leu113 and the effect of Leu113Ser mutation. The residues shown are, NT-4-Chain A: Y176, W192; NT-4-Chain B: W103' and TrkB: T352 and M354. **A**: L113 in ball and stick showing the orientation and hydrophobic cluster around Leu113. **B**: Ser113 in ball and stick showing the gap formed by L113S mutation and the orientation of serine away from hydrophobic cluster.

According to the known crystal structure of the NT-4-TrkB complex Leu113 is located near the surface of NT-4 (Figure 2 and Figure 3A) but in the hydrophobic core comprising residues from both the chains of NT-4 and a chain of TrkB. The modeled structure of Leu113Ser showed a gap (Figure 3B) compared to the wild type structure due to the substitution with the much smaller polar amino acid. The hydrophilic nature of the serine residue is also likely to cause destabilization of the hydrophobic core due to its orientation towards the solvent. As a result, it is highly likely that Leu113Ser disrupt the integrity of both tertiary and quaternary structure of the protein complex leading to disruption of the binding with TrkB and thus its activation.

## Discussion

In this study we identified a novel mutation (Leu113Ser) in *NTF4* in our analysis of 174 POAG patients of Chinese ethnicity. Pathogenicity of this mutation is strongly supported by the fact that this change was not identified in nearly 180 control chromosomes as well by the results of our molecular modeling. The structural analysis indicated that the non-conservative substitution of this highly conserved, hydrophobic residue with a strongly hydrophilic residue is likely to drastically alter the NT-4 protein structure near the TrkB binding site and disrupt the formation of the NT-4-TrkB complex required for the activation of TrkB. However, it should be noted that pathogenicity of this mutation can only be proven conclusively through in vitro functional analysis of the mutant protein.

We did not identify any of the *NTF4* mutations previously reported in European POAG patients in our Chinese cohort, including the most frequent mutation R206W, suggesting that *NTF4* disease-causing mutations are ethnic specific. We also did not find this gene to be very polymorphic in the Chinese. Moreover, our findings of only a single mutation indicate that *NTF4* mutations are a rare cause of POAG in the Chinese. Recently an investigation of the *NTF4* gene in 443 POAG patients of European ancestry in the south-eastern United States found several of the risk alleles previously identified by Pasutto et al. [17] not only in the case population, but also among the control group, thus casting doubt over the involvement of *NT-4* in POAG causation [25]. However, the control group used by Liu and coworkers [25] was younger than that used by the original study; therefore the possibility remains that some of these controls in whom ‘mutations’ were identified may develop the disease at a later date. Analysis of *NTF4* in several more POAG cohorts of different ethnicities, such as our study, is needed to clarify the involvement of *NTF4* in POAG.

Previous candidate gene analyses done by other groups of known POAG genes have reported lower incidence of *MYOC* and *OPTN* mutations in Chinese POAG cohorts compared to Caucasian patient cohorts [26-28]. The latest POAG gene, *NTF4*, accounted for 1.7% of POAG in Caucasians but only 0.6% (95% Confidence Interval: 0.02 to 3.16%) in our Chinese cohort, which again reflect ethnic variations in mutation frequencies found for POAG genes. However, mutations in the three known POAG genes (*MYOC*, *OPTN*, and *WDR36*) account for no more than 10% of POAG patients in any ethnicity. This, with the added rarity of *NTF4* mutations supports the case that POAG is more a complex genetic disorder, with mutations within single genes contributing only a small fraction toward its etiology.

Interestingly, the patient identified with the Leu113Ser mutation had unilateral glaucoma. It is possible that the fellow eye, although not affected at present, may develop glaucoma at a later date. Unilaterality observed in our POAG patient may also suggest that for this particular germ line mutation in *NTF4,* the mutation alone is not sufficient and that additional localized environmental factors are required to produce the disease. In this case, we speculate that the increased IOP in the affected eye is the most likely insult required to cause cell death in ganglion cells with sub-optimal neuro-protection due to the mutation in *NTF4*. The different IOPs in the two eyes could reflect differences between the two sides in ocular development that may have resulted in altered outflow pathway mechanics and IOP regulation. The Leu113Ser mutation was identified in a patient with no recorded family history of POAG, and may therefore represent a denovo pathogenic mutation likely to segregate in a dominant fashion in subsequent generations. We are yet unable to demonstrate co-segregation of the mutation with disease in the family of this patient. This is mainly due to the non-availability of parental DNA due to death, and also the non-compliance of some family members for genetic analysis. We hope to follow up on family members of this patient for future clinical and DNA analysis.

*NTF4*, a member of the neurotrophin protein family which are concerned with the survival of neurons including RGCs, is executed by the phosphorylation of TrkB receptors. Based on their findings of the association between mutations in *NTF4* and POAG, Pasutto and coworkers [17] have demonstrated an impaired TrkB activation as a possible pathway in the pathophysiology of glaucoma. The identification of mutations in *NTF4* also suggests that other members of this neutrophin protein family and its receptors are possible good candidates to study for their involvement in the pathogenesis of POAG.

In conclusion, we have identified a novel mutation in *NTF4* that provides further evidence that impaired neurotrophin signaling or compromised trophic support to the retina may underlie ganglion cell death in POAG. This study indicates that mutations in *NTF4* only account for ≤1% of Chinese POAG patients and is therefore a rare cause of POAG in the Chinese. However, given the recent contradictory findings for *NTF4*, the screening of *NTF4* in POAG patients of other ethnicities and meta-analyses of different studies in POAG patients are required to establish the extent of involvement of *NTF4* in glaucoma.
